# Impact Resistance Study of Fiber–Metal Hybrid Composite Laminate Structures: Experiment and Simulation

**DOI:** 10.3390/ma18122906

**Published:** 2025-06-19

**Authors:** Zheyi Zhang, Haotian Guo, Yang Lan, Libin Zhao

**Affiliations:** 1 School of Mechanical Engineering, Hebei University of Technology, Tianjin 300130, China; zyzhang@hebut.edu.cn (Z.Z.); 202321202107@stu.hebut.edu.cn (H.G.); 202331205125@stu.hebut.edu.cn (Y.L.); 2 Key Laboratory of Advanced Intelligent Protective Equipment Technology, Ministry of Education, Tianjin 300401, China; 3 Key Laboratory of Hebei Province on Scale-Span Intelligent Equipment Technology, Hebei University of Technology, Tianjin 300401, China

**Keywords:** thermoplastic, fiber–metal laminates, impact, finite element simulation

## Abstract

Thermoplastic carbon fiber/aluminum alloy hybrid composite laminates fully integrate the advantages of fiber-reinforced composites and metallic materials, exhibiting high fatigue resistance and impact resistance, with broad applications in fields such as national defense, aerospace, automotive engineering, and marine engineering. In this paper, thermoplastic carbon fiber/aluminum alloy hybrid composite laminates were first prepared using a hot-press machine; then, high-velocity impact tests were conducted on the specimens using a first-stage light gas gun test system. Comparative experimental analyses were performed to evaluate the energy absorption performance of laminates with different ply thicknesses and layup configurations. High-speed cameras and finite element analysis software were employed to analyze the failure process and modes of the laminates under impact loading. The results demonstrate that fiber–metal laminates exhibit higher specific energy absorption than carbon fiber composite laminates. Meanwhile, the numerical simulation results can effectively reflect the experimental outcomes in terms of the velocity–time relationship, failure modes during the laminate impact process, and failure patterns after the laminate impact.

## 1. Introduction

Fiber–metal laminates (FMLs), fabricated by alternately bonding high-strength metal sheets with fiber-reinforced composite laminates, synergistically combine the advantages of metals and composites, achieving superior integrated properties unattainable by individual constituents [1,2]. These hybrid structures exhibit exceptional specific strength, corrosion resistance, fatigue durability, damage tolerance, notch sensitivity resistance, impact resistance, and design flexibility. In the defense sector, FMLs are predominantly utilized in ballistic protection systems due to their outstanding impact resistance [3,4]. Their applications extend to energy-absorbing structures for vehicle crash mitigation and armor systems in naval vessels [5,6,7]. Furthermore, FMLs serve as critical components in aircraft skins and fuselage structures [8], exemplified by their application in the Airbus A380 fuselage structure. However, such structures are subjected to external impact threats during service, including bird strikes and hail impacts. According to International Civil Aviation Organization statistics, over 20,000 bird strike incidents occur annually worldwide, underscoring the significance of impact safety in aviation operations [9,10].

FMLs exhibit complex anisotropic mechanical properties, with their structural performance governed by multiple parameters. The dynamic failure mechanisms and energy dissipation behaviors of FMLs demonstrate significant rate-dependent characteristics under varying impact velocities. When subjected to high-velocity impact loading, the dynamic response of FMLs involves nonlinear phenomena including large geometric deformation, fiber/metal fracture, and interfacial damage. Systematic characterization of these dynamic behaviors and failure mechanisms forms the fundamental basis for protective structure design. Thermoplastic resin-based FMLs have attracted growing attention in defense and aerospace applications due to their elevated-temperature resistance, fatigue resistance, and impact resistance. The impact resistance, as a critical advantage of thermoplastic resin-based FMLs, has drawn significant research interest, though related investigations remain limited. Abdullah et al. [11] investigated the high-velocity impact performance of polypropylene-based FMLs with different configurations and constituent ratios. Their results indicated that 2/1 FMLs with thicker polypropylene-based fiber-reinforced composite layers exhibited the highest specific penetration energy, while multi-layered FMLs (e.g., 4/3 and 3/2 configurations) maintained approximately constant specific penetration energy with increasing metal layer count. Ferrante et al. [12] conducted impact tests on basalt fiber-reinforced FMLs using hemispherical and elliptical projectiles, demonstrating the superior performance of basalt FMLs over monolithic aluminum plates under both impact scenarios. Fatt et al. [13] performed ballistic impact experiments on GLARE laminates, revealing a 15% increase in ballistic limit compared to bare 2024 aluminum through epoxy matrix composite integration. Li et al. [14] conducted a comparative study on the impact resistance of titanium alloy laminates reinforced with carbon fiber and ultra-high-molecular-weight polyethylene (UHMWPE) fibers. Their findings demonstrated that incorporating UHMWPE fiber layers into FML sandwich composites significantly enhances ballistic performance and energy absorption capacity. Gao et al. [15] performed high-velocity impact tests to investigate the ballistic and delamination mechanisms of CFRP and CFRP-based FMLs. The results revealed that FMLs with equivalent thickness exhibit higher ballistic limits and superior impact resistance compared to monolithic CFRP laminates. Additionally, the energy absorption process displayed a distinct bilinear tendency with increasing impact energy. Delamination predominantly initiated at adjacent layers with dissimilar materials or ply orientations, where interlaminar shear stresses and through-thickness bending moments promoted crack propagation. Polyetheretherketone (PEEK) thermoplastic-based FMLs exhibit superior impact resistance; however, research on their high-velocity impact response characteristics and failure mechanisms remains in the nascent stage. Furthermore, structural design references for thermoplastic resin-based FMLs are limited, necessitating further exploration into rational design approaches for enhancing laminate impact resistance. This study employed high-speed photography during high-velocity impact testing to capture dynamic failure processes and analyze failure mechanisms, revealing the failure progression of PEEK-based FMLs under varying impact velocities.

With the rapid advancement of commercial finite element software, numerical simulation techniques have been extensively employed to investigate structural failure and damage in FMLs, facilitating the interpretation of non-quantifiable phenomena observed in experimental studies. Furthermore, numerical modeling circumvents the need for repetitive and time-consuming experimental procedures while providing substantial datasets for optimization design of FMLs. Li et al. [16] developed a finite element model for FMLs incorporating viscoelastic interlayers. Through combined impact–vibration tests, they identified that maintaining an optimal viscoelastic layer-to-FML thickness ratio ranging between 30% and 40% ensures superior impact resistance and vibration damping performance. Yang et al. [17] conducted high-velocity bird strike simulations on FMLs, revealing linear correlations between impact velocity and both delamination area and matrix damage area. The energy absorption mechanisms were found to exhibit three dominant modes: fiber fracture, matrix damage, and interfacial delamination. Yaghoubi et al. [18] established an LS-DYNA finite element model demonstrating strong agreement between simulation and experimental results in terms of impact velocity versus residual velocity relationships, damage morphology, and projectile residual length. The transient strain profiles from simulations matched experimental measurements, thereby validating the model’s accuracy. Numerical analysis further indicated that 6/5 FMLs (6 metal layers to 5 composite layers) achieved the highest maximum contact force, while 2/1 FMLs exhibited the lowest. Below the ballistic limit velocity, maximum contact force increased proportionally with projectile velocity, whereas beyond this critical threshold, contact force became insensitive to further velocity increments.

In summary, research on the high-velocity impact response characteristics and failure mechanisms of thermoplastic resin-based FMLs remains at an early stage. To investigate their impact resistance, this study fabricated thermoplastic carbon fiber-reinforced composite laminates and thermoplastic resin-based FMLs using hot-press molding technology. A specialized fixture with three-edge fixed and one-edge free clamping was designed to ensure high-speed camera clarity without influencing specimen failure modes or impact performance. The dynamic responses and failure mechanisms of both laminates were comparatively analyzed through single-stage high-velocity impact tests. Given the limited simulation studies on thermoplastic carbon fiber/aluminum hybrid composite laminates under high-velocity impact, particularly regarding systematic investigations of stacking sequence and impact energy effects on failure modes and energy absorption, this work established a nonlinear finite element model in LS-DYNA R11 software. The model incorporated a series of constitutive criteria including the Johnson–Cook model for metals and Hashin’s failure criterion or the Chang–Chang failure criterion for composites. By validating against experimental results, primary failure modes of FMLs were elucidated and the finite element model’s effectiveness was verified. These findings provide technical support and reference for designing advanced protective structures.

## 2. Materials and Methods

### 2.1. Specimen Preparation

#### 2.1.1. Test Materials and Pretreatment

The preparation of thermoplastic resin-based fiber–metal hybrid composite laminates requires consideration of the characteristics of both fiber and metal materials to enhance the interfacial strength between them and ensure that the metal does not undergo plastic deformation during the preparation process.

The aluminum alloy used in this study was a 2024-T3 model, provided by Southwest Aluminum (Group) Co., Ltd. (Chongqing, China), with a density of 2.78 g/cm^3^, Young’s modulus of 73 GPa, Poisson’s ratio of 0.31, yield strength of 310 MPa, and tensile strength of 456.4 MPa. Prolonged exposure to the natural environment causes the formation of a dense oxide layer on the aluminum alloy surface, which can affect the interfacial bonding strength between fiber and metal in the fiber–metal hybrid composite laminates. Therefore, before the preparation, the aluminum alloy surface was abraded with 180-grit sandpaper prior to fabrication [19,20] and cleaned with acetone and water separately to remove the oxide film while increasing the surface roughness and the bonding area.

The T700-grade carbon fiber-reinforced PEEK prepreg tape used in this paper was provided by Shandong Node New Material Technology Co., Ltd. (Dezhou, China). The thickness of a single layer is 0.13 mm. Its main performance parameters are shown in Table 1. In the process of preparing fiber–metal hybrid composite laminates, the layering mode and thickness can be flexibly designed.

When the fiber–metal hybrid composite laminates are subjected to external impact, the interface between the fiber and the metal is prone to delamination failure. Additionally, the thermal expansion coefficients of the two materials are different, making the interface relatively weak. To improve this issue and reinforce the interfacial strength, resin film is placed at the interface. The resin film used in this paper is PEEK film, with a single-layer thickness of 0.1 mm.

Surface oil and impurities need to be removed before preparation to prevent them from affecting the interfacial strength between the prepreg tape and polyetheretherketone film. The carbon fiber prepreg material and film were rinsed twice with acetone solution. Then, they were rinsed with water and wiped dry, before being put in the oven to dry completely.

#### 2.1.2. Laminate Preparation Process

Fiber–metal hybrid composite laminates are primarily used in impact-resistant structures. Currently, the forming processes mainly include hand layup, autoclave, and hot molding. Considering that the forming temperature of thermoplastic fiber–metal hybrid composite laminates is relatively high, and this paper does not involve complex shape research, the traditional autoclave process is not applicable. Hot molding refers to placing the fiber prepreg and metal on the mold of a hot press after cutting and laying. Under precise pressure and temperature control, the semi-cured resin is cooled and solidified after flowing. The specimens in this study were prepared using the hot-molding process, which can ensure better processing accuracy and quality.

**Figure 1:**
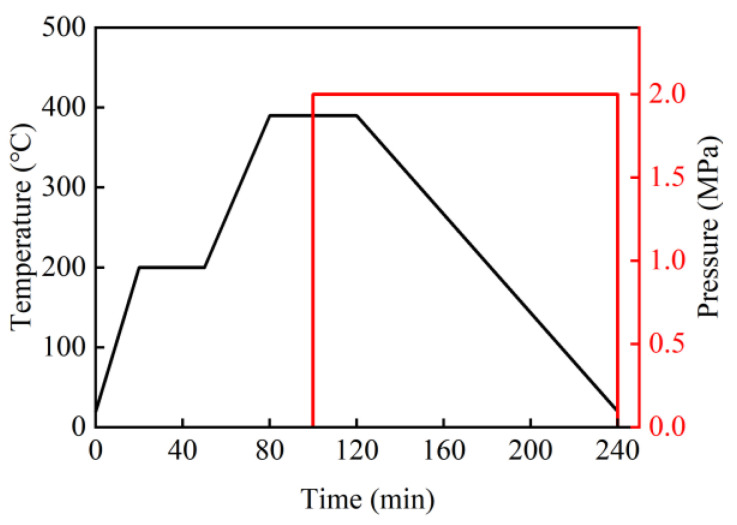
Forming process of PEEK-based composite laminates.

The specimens were prepared with dimensions of 75 × 75 mm, using a 1 mm thick aluminum alloy plate. To provide a reference for comparison, thermoplastic carbon fiber-reinforced composite laminates and thermoplastic carbon fiber/aluminum alloy hybrid composite laminates were also prepared, as shown in Table 2. Firstly, the aluminum alloy material, carbon fiber-reinforced PEEK prepreg tape, and PEEK film were cut to the size of 100 × 100 mm using a cutting machine, and the ply angle was selected as 0° and 90°, which was convenient for precise control in the preparation process and resulted in better mechanical properties. Before preparation, each component material was cleaned, dried, and laminated according to the designed structure. Fiber–metal hybrid composite laminates are prone to delamination failure at the fiber–metal interface under external impact, with the thermal expansion coefficient mismatch between materials contributing to interface vulnerability. To fabricate laminates with superior integrated mechanical properties, resin films were interleaved at interfaces to enhance interlaminar strength. The resin film employed in this study was a PEEK film with a single-ply thickness of 0.1 mm. During specimen processing, three adhesive film layers were utilized to achieve full interfacial bonding between materials [29,30]. When the resin sufficiently wets the metal surface, the mechanical interlocking performance of the metal surface can be fully utilized. Therefore, the PEEK film should have good fluidity during processing. The hot-molding forming process is shown in Figure 1, which includes the following main steps: (1) Preheating: To ensure uniform heating of all components and facilitate demolding, the mold was preheated to 200 °C. Then, it was put into the laid laminated structure and kept there for 30 min. (2) Heating up: The temperature of the upper and lower molds was set to 390 °C; the laminated structure was allowed to heat up with the mold and was kept at this temperature for 15 min. (3) Pressing: After the material temperature was balanced, 2 MPa pressure was applied, and it was kept at this pressure for 15 min. (4) Pressure maintaining cooling: The pressure stopped increasing, and the mold was allowed to cool to room temperature while maintaining pressure.

**Figure 2:**
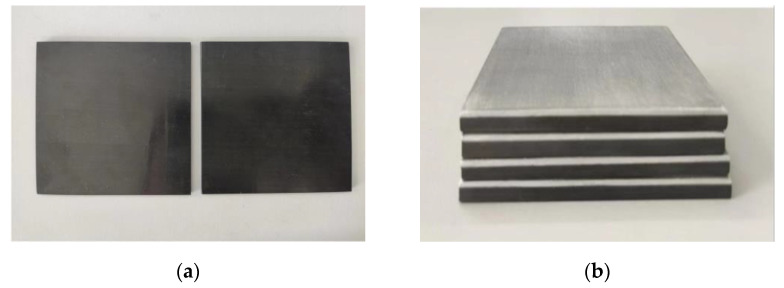
(**a**) Thermoplastic carbon fiber-reinforced composite laminates. (**b**) Thermoplastic carbon fiber/aluminum alloy hybrid composite laminates.

After the mold temperature had cooled to room temperature, the specimen was removed. Then, the resin and burrs that had spilled over the edges of the laminated boards were removed. Then, it was cut to a size of 75 × 75 mm according to the fixture size of the high-speed impact equipment. The finished specimen is shown in Figure 2.

### 2.2. High-Speed Impact Test System

**Figure 3:**
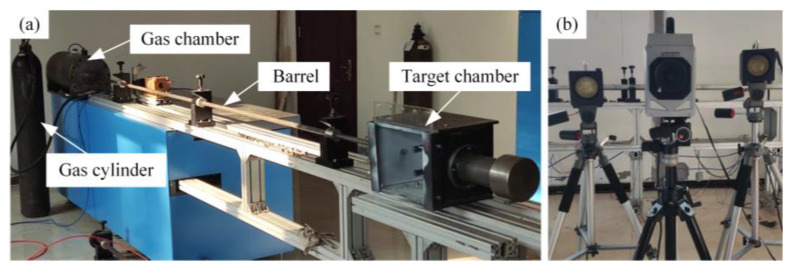
First-level light gas gun equipment and high-speed camera: (**a**) First-level light gas gun experimental system and (**b**) Photron high-speed camera.

High-speed impact tests are conducted on a one-stage light gas gun experimental system, which consists of an external gas cylinder, a gas chamber, valves, a barrel, a projectile, a target chamber, fixtures, and a high-speed camera; the high-pressure gas medium was compressed air, with a launch pressure below 10 MPa and a maximum velocity of 300 m/s. The ambient atmospheric pressure ranged from 86.0 to 106.0 kPa, with relative humidity below 85% RH. The experimental setup is shown in Figure 3. The tests used a spherical projectile with a diameter of 9.5 mm, and its material grade was SUJ2. The instantaneous release of compressed gas in the gas chamber propels the projectile at high speed. The control of the projectile’s speed is achieved indirectly by adjusting the internal pressure of the gas chamber; thus, multiple test shots are required before the experiment to achieve the desired speed range.

**Figure 4:**
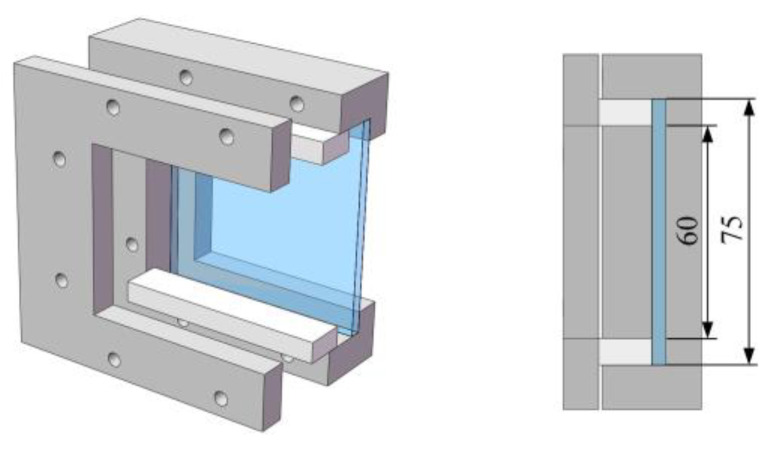
Test fixture.

The test fixture was placed in the target chamber to avoid any danger caused by debris generated during high-speed impact. To study the dynamic impact response of protective materials in high-speed scenarios, it was necessary to avoid interference from the fixture with the high-speed camera’s recording, which could affect the camera to record the failure mode of the target plate. The design of the fixture is shown in Figure 4, and the boundary conditions of three fixed ends and one free end can be achieved for the target plate. Since this study focuses on the primary failure mechanisms and impact resistance of specimens under high-speed impact rather than absolute engineering parameters (e.g., precise peak load, full-scale structural bearing capacity), the influence of boundary conditions with three fixed edges and one free edge is deemed negligible for the present research outcomes.

The high-speed camera was used to collect the projectile velocity during the test and record the failure mode of the target plate. The test used a Photron high-speed camera (Shanghai, China), as shown in Figure 3b. The experimental process was recorded using an illumination system at a full-frame resolution of 256 × 32 pixels and an imaging rate of 80,000 fps.

## 3. Results and Discussion

### 3.1. Test Result Analysis

#### 3.1.1. High-Speed Impact Test Results

The high-speed impact test data for different types of laminates is shown in Table 3. Because composite laminates are anisotropic materials, in order to compare the impact resistance of laminates with different thicknesses and masses, energy absorption, specific energy absorption, and energy absorption per unit thickness are used to compare the performance of laminates. The specific calculation formulas are as follows:

1. Total energy absorption $E_{a}$:
  (1) $$E_{a}=\;\frac{1}{2}m_{F}\left({v_{i}}^{2}\;-\;{v_{r}}^{2}\right),$$
  where $m_{F}$ is the projectile mass; $v_{i}$ is the impact velocity; and $v_{r}$ is the residual velocity.
2. Specific energy absorption *Q*:
  (2) $$Q=\;\frac{E_{a}}{m},$$
  where m is laminated quality.
3. Energy absorption per unit thickness *e*:
  (3) $$e=\frac{E_{a}}{h},$$
  where h is the laminate thickness.

For the same type of laminate, it can be observed from the test that the energy absorption of the laminate increases with the increase in the impact velocity of the projectile. This indicates that the impact resistance of composite laminates increases with the increase in projectile impact velocity. In the range of 200 m/s, the difference between [0/90/90/0]_6_ and [0/90/90/0]_5_ specific energy absorption and unit thickness is small, which indicates that the impact resistance of carbon fiber laminates can be improved by increasing the thickness of carbon fiber laminates, but the efficiency is not high. For different types of laminates, in the speed range of 200 m/s, the specific energy absorption of [0/90/90/0]_5_/Al is 3.7% higher than that of [0/90/90/0]_5_, and the energy absorption per unit thickness is increased by 24.4%. This shows that the hybrid method of fiber and metal has a better effect on improving the impact resistance of composite laminates, which can significantly improve the energy absorption per unit thickness and reduce the thickness of laminates. The [0/90/90/0]_6_/Al specimen was not penetrated when subjected to impact at 200.4 m/s. Fiber plies exhibited fracture and delamination, while the metal layer developed prominent bulging without perforation. Ballistic limit failure was not achieved, with nearly complete absorption of impact energy. Subsequent data from this test series are used exclusively for damage mechanism analysis. Therefore, carbon fiber/aluminum alloy hybrid composite laminates have excellent impact resistance, especially in improving the thickness of composite laminates with a wide range of design potential. The ply angle and thickness ratio of carbon fiber need to be further explored.

#### 3.1.2. Dynamic Response Analysis

Different types of laminates have different dynamic impact response processes, which in turn produce different failure modes. It is of great significance to study and analyze the dynamic impact response process and failure mode of different types of laminates to guide the structural design of laminates. In order to compare the dynamic impact response of different types of laminates, a high-speed camera was used to record the process of projectile penetration of the laminates. The high-velocity impact process diagram assumes that the projectile is close to the laminate at a time of 0 μs to illustrate the penetration process of the laminate.

**Figure 5:**
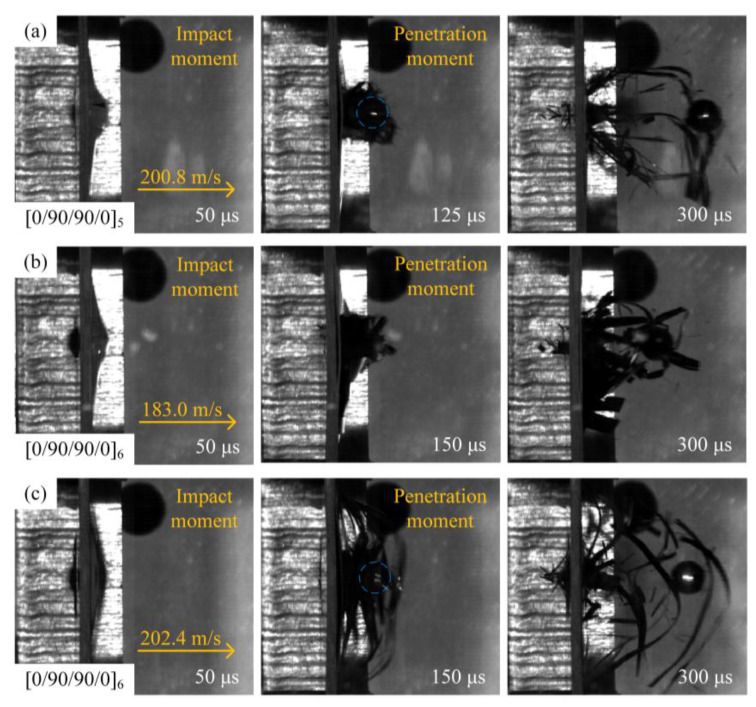
High-speed impact of spherical projectiles on different types of laminates: (**a**) projectile impacts [0/90/90/0]_5_ at 200.8 m/s; (**b**) projectile impacts [0/90/90/0]_6_ at 183.0 m/s; and (**c**) projectile impacts [0/90/90/0]_6_ at 202.4 m/s.

Figure 5 shows the high-velocity impact process of the [0/90/90/0]_5_ and [0/90/90/0]_6_ laminates recorded by a high-speed camera. Taking the process diagram of the [0/90/90/0]_5_-type laminate under the impact of a 200.8 m/s spherical projectile as an example, the dynamic impact response process of the laminate is illustrated. As shown in Figure 5a, the spherical projectile penetrated the entire laminate, and at 50 μs, the projectile penetrated the interior of the laminate, resulting in a local bulge on the back of the laminate and a break in some of the fibers. At 125 μs, the projectile completely penetrated the laminate, and under the compression and tension of the projectile, the fiber bundles at the protrusion on the back of the laminate began to detach from the laminate, and a small number of fiber breakages could be observed around the protrusion. At 300 μs, the projectile continued to advance, accompanied by more fiber bundles detaching from the back of the laminate and a large amount of fiber debris flying out of the front and back of the laminate. When the composite laminate thickness increases, as shown in Figure 5c, the blue circle indicates the position of the spherical projectile, at 50 μs, the [0/90/90/0]_6_ laminate produced a larger back bulge than the [0/90/90/0]_5_-type laminate shown in Figure 5a. The convex laminate more fully absorbed the impact energy of the projectile.

At 150 μs, the projectile completely penetrated the laminate, and the residual velocity of the projectile decreased significantly compared to Figure 5a, indicating that the impact resistance of the [0/90/90/0]_6_ laminate was enhanced. When the impact velocity of the projectile decreased, as shown in Figure 5b, the time for the projectile to penetrate the laminate increased, and the damage to the laminate decreased.

**Figure 6:**
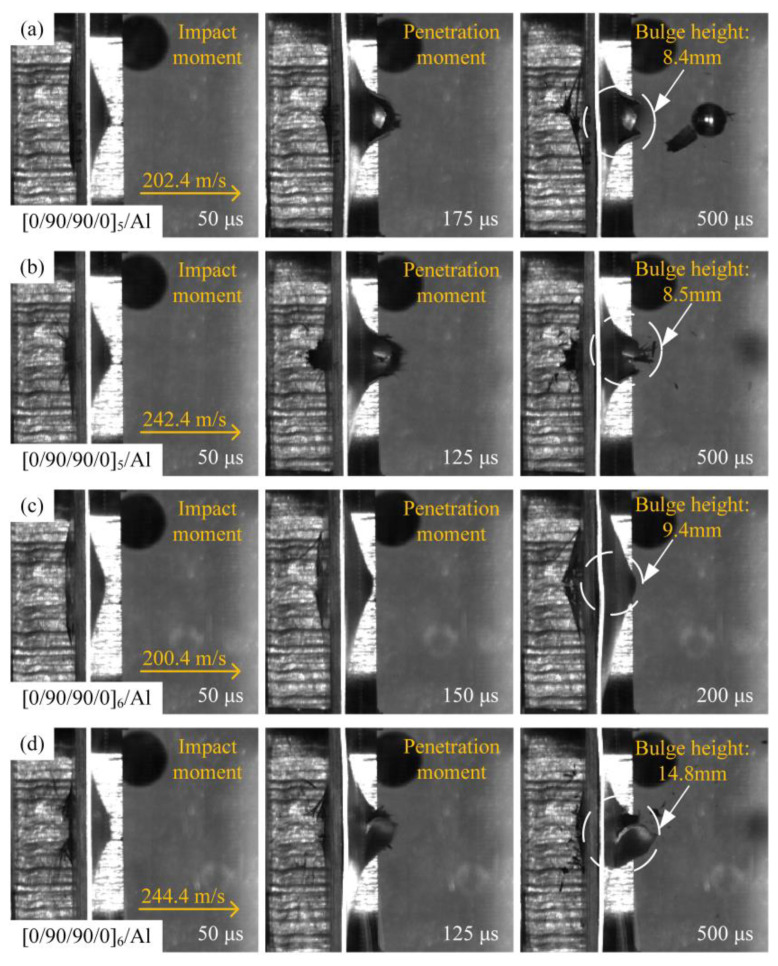
High-speed impact of spherical projectiles on different types of metal-hybrid laminate: (**a**) projectile impacts [0/90/90/0]_5_/Al at 202.4 m/s; (**b**) projectile impacts [0/90/90/0]_5_/Al at 242.4 m/s; (**c**) projectile impacts [0/90/90/0]_6_/Al at 200.4 m/s; and (**d**) projectile impacts [0/90/90/0]_6_/Al at 244.4 m/s.

Figure 6 shows the high-velocity impact process diagram of the [0/90/90/0]_5_/Al and [0/90/90/0]_6_/Al laminates recorded by a high-speed camera. The process of the [0/90/90/0]_5_/Al-type laminate under the impact of a spherical projectile of 202.4 m/s is shown in Figure 6a. The projectile first came into contact with a layer of carbon fiber-reinforced composite. At 50 μs, the projectile completely invaded the laminate. The local fiber bundles on the front surface of the laminate were slightly bulged by the projectile, and the aluminum alloy layer on the back of the laminate was subjected to large plastic deformation. At 175 μs, the projectile was about to penetrate the entire laminate. Under the tensile and shear action of the projectile, the plastic deformation of the aluminum alloy layer on the back of the laminate continued to increase, and cracks appeared. At 300 μs, the projectile completely penetrated the entire laminate, and the aluminum layer on the back of the laminate was torn off by the projectile to become petal-shaped, unlike Figure 5, which is accompanied by carbon fiber powder and a small amount of pulled fiber bundles due to the limitation of the aluminum alloy layer. With the projectile velocity increased, as shown in Figure 6b, cracks appeared in the aluminum alloy layer on the back of the laminate at 125 μs. The time for the projectile to penetrate the laminate was affected by the projectile velocity compared to Figure 6a, and the time for the laminate to undergo damage was significantly shorter. When the thickness of the carbon fiber-reinforced composite layer increases, as shown in Figure 6c, under the impact of the projectile at 200.4 m/s, the [0/90/90/0]_6_/Al laminate was not penetrated, and the plastic deformation of the aluminum alloy layer on the back side was more serious than that of other types of laminates. As shown in Figure 6d, at 125 μs, the laminate had a large crack and the petal-shaped fracture was not obvious, which was due to the fact that the thicker carbon fiber-reinforced composite layer bulges more during the impact process, which increased its contact area with the aluminum alloy layer, resulting in the enhanced shear effect of the projectile. So the increase in the thickness of the fiber ply changed the failure mode and failure degree of the laminate.

#### 3.1.3. Failure Morphology Analysis

**Figure 7:**
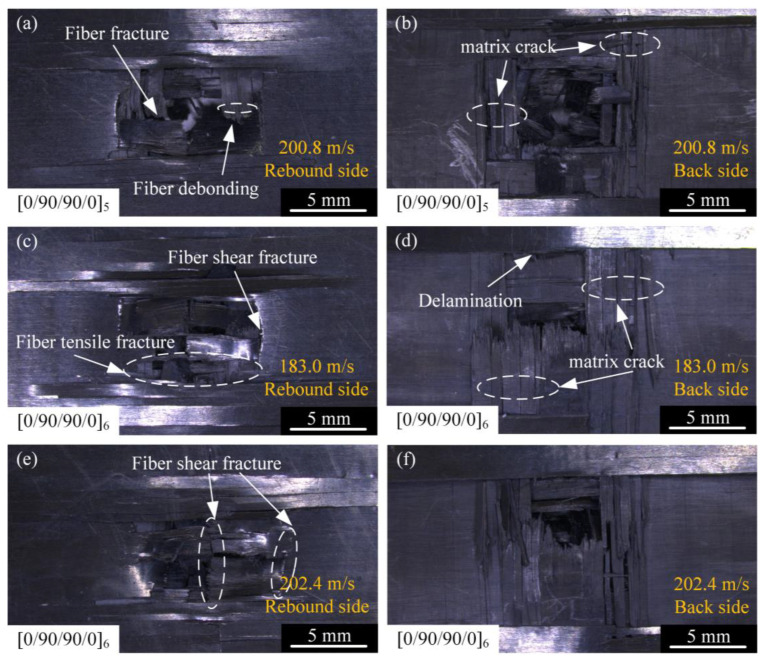
Damage morphologies of [0/90/90/0]_5-_ and [0/90/90/0]_6_-type laminates on the front and back surfaces: (**a**) projectile impacts [0/90/90/0]_5_ at 200.8 m/s, rebound side; (**b**) projectile impacts [0/90/90/0]_5_ at 200.8 m/s, back side; (**c**) projectile impacts [0/90/90/0]_6_ at 183.0 m/s, rebound side; (**d**) projectile impacts [0/90/90/0]_6_ at 183.0 m/s, back side; (**e**) projectile impacts [0/90/90/0]_6_ at 202.4 m/s, rebound side; and (**f**) projectile impacts [0/90/90/0]_6_ at 202.4 m/s, back side.

Different types of laminates were affected differently by the impact of the projectile at high speed, and the failure mechanism can be analyzed according to the failure morphology of the laminates. The failure morphology of the front and back of the [0/90/90/0]_5_ and [0/90/90/0]_6_ types of laminates after being impacted by the projectile at high speed is shown in Figure 7.

**Figure 8:**
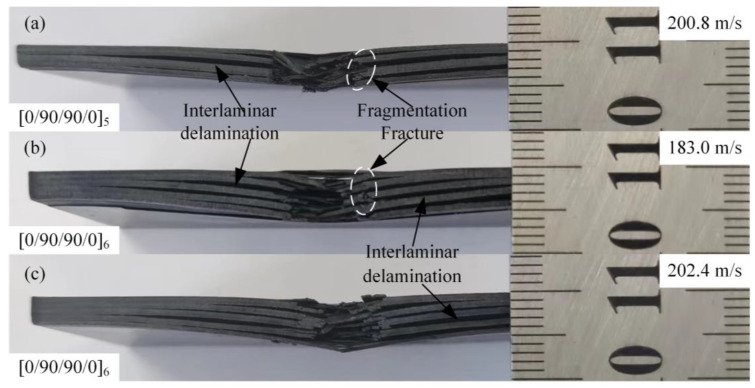
Damage morphologies of [0/90/90/0]_5_- and [0/90/90/0]_6_-type laminates on the cross-section: (**a**) projectile impacts [0/90/90/0]_5_ at 200.8 m/s, damage morphology; (**b**) projectile impacts [0/90/90/0]_6_ at 183.0 m/s, damage morphology; and (**c**) projectile impacts [0/90/90/0]_6_ at 202.4 m/s, damage morphology.

The failure morphology of the cross-section is shown in Figure 8. It can be seen that under the high-speed impact of the projectile, the laminate was broken down. Under the action of shear and tensile load, the failure mode of the front of the laminate was fiber debonding, fiber fracture, and matrix crack along the fiber direction. Fiber debonding usually begins at a stress level below the strength of the matrix and fiber, propagates along the interface between the fiber and the matrix, and further forms larger cracks in the matrix material. Fiber debonding absorbs a certain amount of energy, while relative sliding between fibers and the matrix dissipates additional energy through interfacial friction. However, fiber debonding compromises the synergistic load-bearing capacity of fibers and the matrix, significantly reducing the stiffness and strength of the composite layer. This process provides additional potential initiation sites for subsequent fiber fracture and markedly promotes the initiation and propagation of delamination [31]. The back of the laminate created a matrix crack that was more pronounced than the rebound side. As shown in Figure 8, according to the analysis of the failure morphology, when the laminate was subjected to the high-velocity impact of the projectile, the shear stress wave generated by the impact reached the inside of the laminate, and the shear stress wave propagated along the direction of the thickness of the laminate. When the shear stress wave propagates to the interface of different media in the laminate, part of the shear stress wave is reflected through the interface to form the propagating tensile stress wave along the in-plane direction, which causes interlayer delamination damage to the laminate. Delamination initiation and propagation require energy dissipation. This phenomenon decouples the originally monolithic thick laminated plate into multiple thinner sub-laminates. These sub-laminates can deform independently and subsequently absorb additional energy through their deformation and mutual friction. While the delaminated regions enable significant plastic deformation in metal layers, delamination severely compromises the global stiffness and bending stiffness of the laminated plate, consequently leading to increased global deformation.

At the same time, under the action of tensile and shear stress, the back side of the laminate shows an obvious outward protrusion, resulting in the detachment of the outermost part of the fiber bundle from the laminate. Fiber fracture usually occurs after matrix cracks and interlaminar delamination, and the extent of fiber fracture is confined to the vicinity of the penetration channel. Fiber fracture represents one of the primary energy absorption mechanisms. This process consumes substantial energy, with fiber pull-out and frictional sliding at fracture interfaces further dissipating energy. However, fiber fracture results in permanent loss of load-bearing capacity. The impact load is subsequently transferred to adjacent fibers, thus potentially triggering cascading fracture. Fiber fracture and matrix crack failure further dominate the resulting composite fracture, as shown in Figure 8. When the shear stress wave propagated to the interface of different media in the laminate, part of the shear stress wave was reflected through the interface to form a propagating tensile stress wave along the in-plane direction, causing delamination damage to the laminate. At the same time, under the action of tensile and shear stress, the back of the laminate had obvious outward protrusions, resulting in the detachment of the outermost part of the fiber bundle from the laminate. Fiber fracture usually occurs after matrix cracks and interlayer delamination, and the range of fiber fracture is limited to the vicinity of the penetration channel. The fiber fracture and matrix crack failure further dominate the composite fracture, as shown in Figure 8.

**Figure 9:**
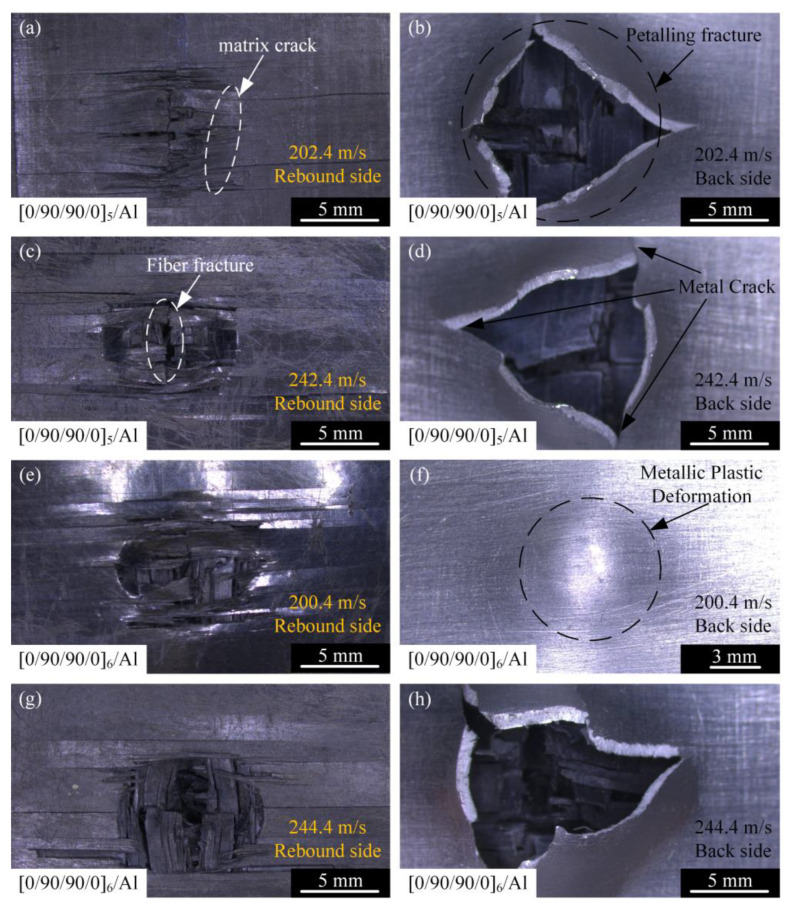
Damage morphologies of [0/90/90/0]_5_/Al- and [0/90/90/0]_6_/Al-type laminates on the front and back surfaces: (**a**) projectile impacts [0/90/90/0]_5_/Al at 202.4 m/s, rebound side; (**b**) projectile impacts [0/90/90/0]_5_/Al at 202.4 m/s, back side; (**c**) projectile impacts [0/90/90/0]_5_/Al at 242.4 m/s, rebound side; (**d**) projectile impacts [0/90/90/0]_5_/Al at 242.4 m/s, back side; (**e**) projectile impacts [0/90/90/0]_6_/Al at 200.4 m/s, rebound side; (**f**) projectile impacts [0/90/90/0]_6_/Al at 200.4 m/s, back side; (**g**) projectile impacts [0/90/90/0]_6_/Al at 244.4 m/s, rebound side; and (**h**) projectile impacts [0/90/90/0]_6_/Al at 244.4 m/s, back side.

**Figure 10:**
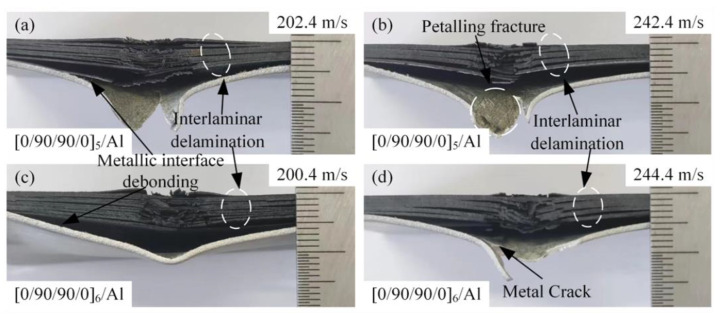
Damage morphologies of [0/90/90/0]_5_/Al- and [0/90/90/0]_6_/Al-type laminates on the cross-section: (**a**) projectile impacts [0/90/90/0]_5_/Al at 202.4 m/s, damage morphology; (**b**) projectile impacts [0/90/90/0]_5_/Al at 242.4 m/s, damage morphology; (**c**) projectile impacts [0/90/90/0]_6_/Al at 200.4 m/s, damage morphology; and (**d**) projectile impacts [0/90/90/0]_6_/Al at 244.4 m/s, damage morphology.

Figure 9 shows the front and back of the [0/90/90/0]_5_/Al and [0/90/90/0]_6_/Al laminates after high-velocity impact. Figure 10 shows the failure morphology of the cross-section after high-velocity impact. In addition to fiber debonding, fiber fracture, matrix cracks along the fiber direction, and delamination between composite layers, there was also fiber–metal interfacial delamination, metal plastic deformation, and petal-shaped fracture. As shown in Figure 9, with the increase in the thickness of the laminate, the damage of the composite material is more serious. Compared with Figure 9a,c,e,g, the fracture degree of the carbon fiber on the front of the laminate is significantly increased. The reason is that when the thickness of the laminate is large, the impact resistance is relatively strong, and the interaction time between the carbon fiber-reinforced composite layer and the projectile is long, so it can more fully dissipate the impact energy. The aluminum alloy layer of the laminate undergoes plastic deformation and petal-shaped fracture under the high-speed impact of the projectile. According to Figure 9b,f, it can be judged that the plastic deformation of the aluminum alloy layer occurred in the early stage of the impact process. The projectile produced a high local force at the contact with the laminate, resulting in partial damage and deformation of the front carbon fiber-reinforced composite layer, and the aluminum alloy layer began to experience bulging and local thinning. Under impact loading, the aluminum alloy layer develops extremely high radial tensile stresses and hoop tensile stresses around the impact zone. Plastic deformation of the metal layer induces local necking, thereby initiating petal-shaped fracture. Due to the extensive deformation area involved in petal-shaped fracture, this mechanism consumes significant energy, and the shape of the petal was related to the velocity of the projectile, as shown in Figure 10. In Figure 10, it can be observed that the laminates were stratified between composites and the fiber–metal interface, and the shear stress waves were transformed into tensile stress waves after sufficient reflection, resulting in lamination failure mainly in the middle and rear of the laminates. At the same time, it can be found in Figure 10 that the fiber fracture degree of the upper part of the laminate was generally less than that of the lower part. Based on this, the aluminum alloy layer in the laminate can improve the energy dissipation of the fiber fracture failure during the impact process. Compared with Figure 10a–d, the delamination failure of laminates was more serious. It can be inferred that the energy dissipation effect of delamination failure of fiber aluminum alloy laminates was more significant in the velocity range close to the ballistic limit.

### 3.2. Numerical Model Validation

#### 3.2.1. Constitutive Relationship and Failure Model for Metal Materials

Johnson–Cook models [32] are often used to describe the behavior of materials at large strains, high strain rates, and high temperatures to analyze and predict the response of materials to dynamic loads. The Johnson–Cook model takes into account the hardening behavior and strain rate effect of the material under dynamic load, and the structure is simple and the parameters are relatively easy to determine. Therefore, it is widely used to simulate impacts, explosions, and other related problems.

The Johnson–Cook model decomposes the equivalent stress into three functions that depend on the strain, strain rate, and temperature degrees. The constitutive relations are as follows:

(4) $$\sigma_{y}=[A+B({\bar{\varepsilon }}^{p}{)}^{n}]\left[1+c\ln\left(\frac{{\dot{\bar{\varepsilon }}}^{p}}{{\dot{\bar{\varepsilon }}}_{0}}\right)\right]\left(1-T^{*m}\right),$$

where $\sigma_{y}$ is the equivalent stress; A is the static yield stress; B is the strain strengthening coefficient; n is the strain strengthening index; c is the strain rate sensitivity coefficient; m is the temperature softening coefficient; ${\bar{\varepsilon }}^{p}$ is the equivalent plastic strain; ${\dot{\bar{\varepsilon }}}^{p}$ is the equivalent plastic strain rate; ${\dot{\bar{\varepsilon }}}_{0}$ is the reference plastic strain rate; and $T^{*}$ is defined as follows:

(5) $$T^{*}={\left(\frac{T\;-T_{r}}{T_{m}-T_{r}}\right)}^{m},$$

where T is the material temperature; $T_{r}$ is room temperature; and $T_{m}$ is the melting point.

In order to determine the structural damage of a material, the failure strain is defined as follows:

(6) $$\varepsilon_{f}=\left[D_{1}+D_{2}\exp\left(D_{3}\sigma^{*}\right)\right]\left[1+D_{4}\ln\left(\frac{{\dot{\bar{\varepsilon }}}^{p}}{{\dot{\bar{\varepsilon }}}_{0}}\right)\right]\left(1+D_{5}T^{*}\right),$$

where $\sigma^{*}$ is the average stress normalized by equivalent stress; $\varepsilon_{f}$ is the equivalent failure strain; and $D_{1}\sim D_{5}$ are the failure constants measured by the test. Assuming that failure occurs when the failure parameter D reaches 1, parameter D is determined by the following formula:

(7) $$D=\;\sum \left(\frac{\Delta {\bar{\varepsilon }}^{p}}{\varepsilon_{f}}\right),$$

where $\Delta {\bar{\varepsilon }}^{p}$ is the equivalent plastic strain increment.

Mechanical properties of materials of 2024-T3 (the density, modulus, Poisson’s ratio, and yield strength) are provided by the manufacturer and other model parameters (the strain strengthening index, the temperature softening coefficient, and the failure constants$\;D_{1}\sim D_{5}$) refer to the relevant literature [33,34,35] and are established after a lot of simulation debugging. Table 4 summarizes the Johnson–Cook model parameters required for 2024-T3 finite element simulations in this paper.

#### 3.2.2. Constitutive Relationship and Failure Model for Composite Materials

Fiber-reinforced composite laminates are usually made up of single-layer panels laid along the laminate thickness direction at a certain angle. Therefore, the single-layer plate is a basic unit of fiber-reinforced composites. The single-layer plate is composed of fibers and matrix, has different mechanical properties along the fiber direction and perpendicular to the fiber direction, and is a typical orthotropic material on the macro scale. The single-layer plate is usually very thin, with a thickness of only 0.1~0.3 mm, and the stress component in the normal direction is much smaller than the stress component in the in-plane direction, which is negligible. So the stress analysis problem of the single-layer plate is usually simplified into a two-dimensional plane stress analysis problem [36], where the reinforced fiber direction of a single-layer plate is defined as one direction or longitudinal and the direction perpendicular to the reinforced fiber is defined as two directions or transverse.

Based on linear elastic mechanics, the constitutive relationship of a single-layer plate is

(8) $$\left[\begin{array}{c} \sigma_{1}\\ \sigma_{2}\\ \tau_{12} \end{array}\right]=\left[\begin{array}{ccc} Q_{11} & Q_{12} & 0\\ Q_{21} & Q_{22} & 0\\ 0 & 0 & Q_{66} \end{array}\right]\left[\begin{array}{c} \varepsilon_{1}\\ \varepsilon_{2}\\ \gamma_{12} \end{array}\right]\;\mathrm{or}\;\left[\begin{array}{c} \varepsilon_{1}\\ \varepsilon_{2}\\ \gamma_{12} \end{array}\right]=\left[\begin{array}{ccc} S_{11} & S_{12} & 0\\ S_{21} & S_{22} & 0\\ 0 & 0 & S_{66} \end{array}\right]\left[\begin{array}{c} \sigma_{1}\\ \sigma_{2}\\ \tau_{12} \end{array}\right],$$

where [Q] is the normal axis stiffness, and [S] is the normal axis flexibility.

The relationship between the elastic constant and the flexibility coefficient is as follows:

(9) $$S_{11}=\frac{1}{E_{1}},\;S_{12}=-\frac{\nu_{12}}{E_{1}}=-\frac{\nu_{21}}{E_{2}},\;S_{22}\;=\frac{1}{E_{2}},\;S_{66}=\frac{1}{G_{12}},$$

where $E_{2}$, $E_{2}$, $G_{12},$ and $\nu_{12}$ are the engineering elastic constants of single-layer plates.

The relationship between the normal axial modulus and the engineering elastic constant is as follows:

(10) $$Q_{11}=cE_{1},\;Q_{12}=c\nu_{12}E_{2}\;=c\nu_{21}E_{1},\;Q_{22}=cE_{2},\;Q_{66}=G_{12},$$

In the formula, the calculation formula of c is as follows:

(11) $$c={\left(1-\nu_{12}\nu_{21}\right)}^{-1}={\left(1-\frac{\nu_{12}^{2}E_{2}}{E_{1}}\right)}^{-1}$$

Under the action of dynamic load, the failure mode of a single-layer plate is complex, and there are four typical failure modes: fiber tensile failure, fiber compression failure, matrix tensile failure, and matrix compression failure. The Chang–Chang failure criterion can fully reflect the four failure modes of single-layer plates and is widely used in finite element simulation. The failure criterion is as follows:

For fiber tensile failure, $\sigma_{11}>0$:

(12) $$e_{f}^{2}=(\frac{\sigma_{11}}{X_{t}}{)}^{2}+\beta (\frac{\sigma_{12}}{S_{c}}{)}^{2}-1,\left\{\begin{array}{c} e_{f}^{2}\;\geq \;0\Rightarrow \mathrm{failed}\\ e_{f}^{2}\;\leq \;0\Rightarrow \mathrm{elastic} \end{array}\right.$$

For fiber compression failure, $\sigma_{11}<0$:

(13) $$e_{c}^{2}=(\frac{\sigma_{11}}{X_{c}}{)}^{2}-1,\;\;\left\{\begin{array}{c} e_{c}^{2}\;\geq \;0\Rightarrow \mathrm{failed}\\ e_{c}^{2}\;\leq \;0\Rightarrow \mathrm{elastic} \end{array}\right.$$

For matrix tensile failure $\sigma_{22}>0$:

(14) $$e_{m}^{2}=(\frac{\sigma_{22}}{Y_{t}}{)}^{2}+(\frac{\sigma_{12}}{S_{c}}{)}^{2}-1,\left\{\begin{array}{c} e_{m}^{2}\;\geq \;0\Rightarrow \mathrm{failed}\\ e_{m}^{2}\;\leq \;0\Rightarrow \mathrm{elastic} \end{array}\right.$$

For matrix compression failure, $\sigma_{22}<0$:

(15) $$e_{d}^{2}={\left(\frac{\sigma_{22}}{2S_{c}}\right)}^{2}+\left[{\left(\frac{Y_{c}}{2S_{c}}\right)}^{2}-1\right]\frac{\sigma_{22}}{Y_{c}}+{\left(\frac{\sigma_{12}}{S_{c}}\right)}^{2}-1,\;\;\left\{\begin{array}{c} e_{d}^{2}\;\geq \;0\Rightarrow \mathrm{failed}\\ e_{d}^{2}\;\leq \;0\Rightarrow \mathrm{elastic} \end{array}\right.,$$

where $X_{t}$ is the longitudinal tensile strength; $X_{c}$ is the longitudinal compressive strength; $Y_{t}$ is the transverse tensile strength; $Y_{c}$ is the transverse compressive strength; and $S_{c}$ is the in-plane shear strength.

Some model parameters of the carbon fiber-reinforced composite material single-layer plate are referenced from the manufacturer’s standard test results (Table 1), while others are referenced from the relevant literature [37,38,39] and established through a large number of simulations and adjustments. Table 5 summarizes the model parameters required for the carbon fiber layer in the finite element simulation of this paper.

#### 3.2.3. Establishment of Nonlinear Finite Element Model

**Figure 11:**
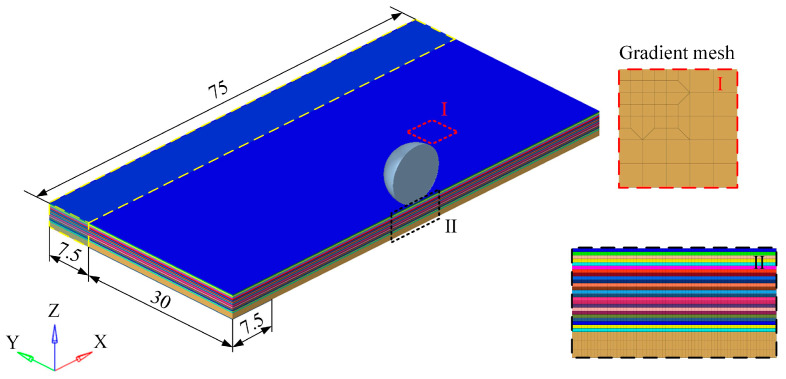
Nonlinear finite element model.

This paper established a finite element model of projectile impact using the finite element analysis software LS-DYNA R11. The detailed structure of the finite element model is shown in Figure 11. The impact direction of the projectile was perpendicular to the laminate, with a diameter of 9.5 mm. The thickness of the carbon fiber single laminate was 0.13 mm, and the thickness of the aluminum alloy layer was 1 mm. According to the symmetry of the model and in order to improve the calculation efficiency, a half model was used to simulate the impact test. The plane size of the model was 75 × 37.5 mm. The geometric model was discretized using hexahedral solid elements. A gradually refined mesh strategy was implemented in the impact zone to enhance both computational efficiency and solution accuracy. Mesh convergence analysis confirmed that the refined central region measured 19.5 × 9.75 mm, with the locally refined elements having an in-plane dimension of 0.125 × 0.125 mm. The geometric model was meshed by hexahedral solid elements, and a gradual refinement meshing strategy for the impact center region was adopted to improve the computational efficiency and accuracy of the model. The size of the refined region in the impact center was 19.5 × 9.75 mm, and the plane size of the refined element was 0.125 × 0.125 mm. The mesh density of the spherical projectile was equivalent to the central mesh density of the target plate to ensure good contact between the target plate and the projectile during the penetration process. In establishing the half-symmetry model, symmetric boundary conditions were applied to the faces at the symmetry planes of the laminate and projectile. To simulate the constraints of the experimental fixture, encastre constraints were imposed along the model edges, specifically on the regions comprising 75 × 7.5 mm and 30 × 7.5 mm elements. The detailed boundary conditions of the numerical model are illustrated in Figure 11.

LS-DYNA incorporates various built-in contact models for use, as selecting a reasonable contact model can effectively improve the computational accuracy of the finite element model. In this study, to avoid the self-penetration of the mesh, the overall model was defined using the contact type ‘CONTACT_AUTOMATIC_SINGLE_SURFACE’. In addition, in the simulation, the unit was deleted when it met the failure criterion. In order to describe the contact between the projectile and the target during the impact process, the ‘CONTACT_ERODING_SURFACE_TO_SURFACE’ model was used, which allowed the contact surface to regenerate after removing the contact unit. The fiber–metal laminates were composed of multi-layer structures, and the layers were bonded by the matrix, which was prone to delamination failure during the impact process. In order to simulate this failure mode, the contact between the carbon fiber single-layer plates and the contact between the carbon fiber and the metal were realized by the ‘CONTACT_AUTOMATIC_SURFACE_TO_SURFACE_TIEBREAK’ contact model. The contact model meant that the nodes on the contact interface adhere to each other in the initial state. Once the failure threshold was reached, the nodes on the contact interface were separated. The layered failure criterion was defined as follows:

(16) $${\left(\frac{\sigma_{n}}{\mathrm{NFLS}}\right)}^{2}+{\left(\frac{\sigma_{s}}{\mathrm{SFLS}}\right)}^{2}\;\geq \;1,$$

where $\sigma_{n}$ and $\sigma_{s}$ represent the normal tensile stress and shear stress of the contact surface, respectively. For NFLS and SFLS, the failure parameters between carbon fiber monolayer plates are 43 MPa and 50 MPa [39]. The interfacial strength between the carbon fiber monolayer and the aluminum alloy is weak, and the failure parameters do not cause a significant difference in impact response [40]. The interface failure parameters between the PEEK-based carbon fiber-reinforced composite and aluminum alloy in the reference are 4.6 MPa and 11.8 MPa, respectively.

A variety of material models were built in LS-DYNA, and the progressive damage model MAT54 was often used in the impact simulation of composite materials. The material model used the Chang–Chang failure criterion to accurately predict various failure modes of composite materials. In the simulation model establishment, the in-plane material directions 1 and 2 of the carbon fiber single-layer plate were aligned with the global X and Y axes, and the material direction 3 along the thickness direction of the laminate was aligned with the global Z axis. For aluminum alloy materials, the Johnson–Cook model MAT15 built-in LS-DYNA was used to accurately simulate the damage characteristics of metal materials. In the high-speed impact test, the spherical projectile of the bearing steel material has almost no deformation. Therefore, the material model MAT20 was used in LS-DYNA to define a rigid body, which can improve the computational efficiency [41].

#### 3.2.4. Finite Element Model Verification

In this paper, the validity of the finite element model was verified by comparing the velocity variation of the projectile, the deformation and failure of the laminate during impact, and the damage morphology of the laminate after impact.

**Figure 12:**
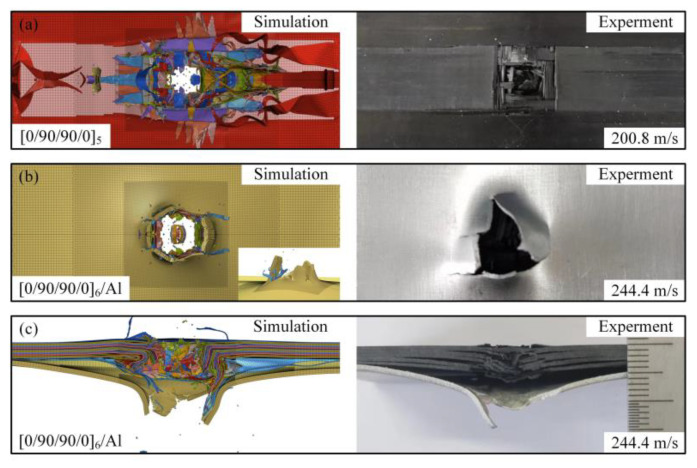
Comparison of simulation and experimental failure mode results: (**a**) projectile impacts [0/90/90/0]_5_ at 202.4 m/s, comparison of simulation and experiment; (**b**) projectile impacts [0/90/90/0]_6_/Al at 244.4 m/s, comparison of simulation and experiment, back side; and (**c**) projectile impacts [0/90/90/0]_6_/Al at 244.4 m/s, comparison of simulation and experiment, lateral side.

Figure 12 shows the comparison of back and cross-sectional damage of the test and simulation results after impact on different types of laminates; the chromatic scheme in the figure denotes distinct ply orientations within the laminates. It can be seen that the [0/90/90/0]_5_-type laminate has similar fiber fracture and fiber bundle detachment on the back surface in the simulation results under the impact of projectiles at 200.8 m/s. And the [0/90/90/0]_6_/Al-type laminate has a petal-shaped fracture and perforation on the back surface of the laminate under the impact of projectiles at 244.4 m/s, which is consistent with the test results. There is a significant interfacial delamination phenomenon near the damage. The damage morphology of the test and simulation results is basically consistent.

**Figure 13:**
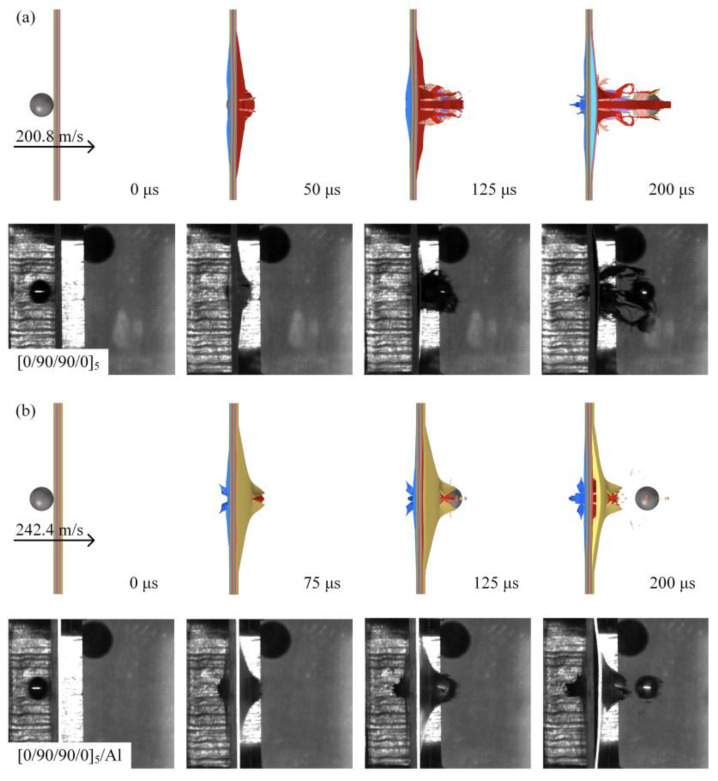
Comparison of simulation and experimental high-speed impact process results: (**a**) projectile impacts [0/90/90/0]_5_ at 202.4 m/s, comparison of impact process simulation and experiment; (**b**) projectile impacts [0/90/90/0]_5_/Al at 202.4 m/s, comparison of impact process simulation and experiment.

High-speed cameras recorded the dynamic images of the projectile impact on the sandwich panel during the test. The finite element model’s calculation results showed that the fiber fracture, backside bulge height, and damage morphology of the sandwich panel at different times were relatively consistent with the test. Figure 13 shows the impact process comparison of specimens of type [0/90/90/0]_5_ and [0/90/90/0]_5_/Al. The displacement of the projectile recorded by the machine in two images and the frame rate of the high-speed camera set in the experiment are used to calculate the speed of the projectile during the impact process, which has been applied in the relevant literature. The velocity of the projectile in the impact process was calculated by the displacement of the projectile recorded by the high speed camera in two images and the frame rate set by the high speed camera in the test. This method has been applied in the relevant literature [42].

The root mean square error (RMSE) is defined as follows:

(17) $$RMSE=\sqrt{\frac{1}{N}\sum_{i=1}^{N}{\left[v_{exp}\left(t_{i}\right)-v_{sim}\left(t_{i}\right)\right]}^{2}}\;$$

where *v*_exp_ represents the ball velocity from the experimental data, and *v*_sim_ denotes the ball velocity from the simulation results. The RMSE is calculated from experimental and simulation data.

**Figure 14:**
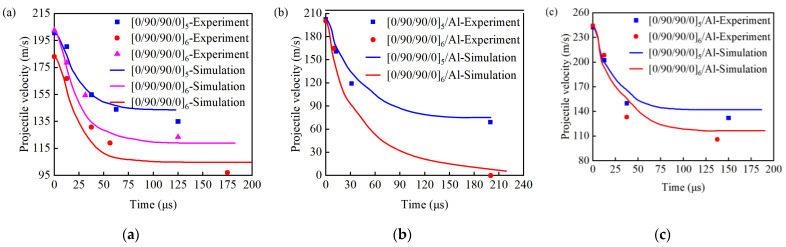
Comparison of projectile velocity changes in simulation and experiment: (**a**) [0/90/90/0]_5_ and [0/90/90/0]_6_; (**b**) [0/90/90/0]_5_/Al and [0/90/90/0]_6_/Al (not penetrated); (**c**) [0/90/90/0]_5_/Al and [0/90/90/0]_6_/Al.

As shown in Figure 14, the speed of the projectile decreases sharply in a short time during the impact process and then tends to stabilize. Comparing the simulation and experimental results, it is found that the projectile velocity–time curve calculated by the finite element model is consistent with the velocity change trend of the projectile in the experiment, with good consistency. Comparing the experimental data and simulation results of the remaining projectile speed greater than 0 m/s, as shown in Table 6, the simulated results exhibited errors within 10%, with the RMSE of the velocity–time curves being less than 5% of the impact velocity. This demonstrates favorable agreement between the model and experiments throughout the impact process, while showing minor deviations from results in the relevant literature, confirming the model’s validity.

In summary, the finite element model established in this paper can accurately simulate the high-speed impact response of different types of laminates from the analysis of projectile velocity change, impact process, and failure mode of the laminates after impact. The finite element model has high accuracy.

## 4. Conclusions

This study introduces the processing and forming process of thermoplastic carbon fiber/aluminum alloy hybrid composite laminates, conducts high-speed impact tests on the specimens, and finally establishes a nonlinear finite element model based on the test details and verifies it. The main conclusions are as follows:

1. According to the processing technology of thermoplastic carbon fiber/aluminum alloy hybrid composite laminates, thermoplastic carbon fiber-reinforced composite laminates and thermoplastic carbon fiber/aluminum alloy hybrid composite laminates were fabricated using a hot-die pressing machine. The process processes are mainly preheating, heating up, pressurizing, and holding pressure cooling.
2. A first-stage light gas gun test system was used to carry out high-speed impact tests on the specimens, and it was found that within the speed range of 200 m/s, the specific energy absorption and energy absorption per unit thickness of [0/90/90/0]_6_ and [0/90/90/0]_5_ had a small difference. The [0/90/90/0]_5_/Al ratio and [0/90/90/0]_5_ specific energy absorption increased by 3.7%, and unit thickness energy absorption increased by 24.4%, suggesting that the use of fiber and metal hybrids could improve the impact resistance of composite materials.
3. The main failure modes of thermoplastic carbon fiber-reinforced composite laminates are fiber debonding, fiber fracture, and matrix cracks along the fiber direction. The failure modes of thermoplastic carbon fiber/aluminum alloy hybrid composite laminates, in addition to fiber debonding, fiber fracture, matrix cracks along the fiber direction, and interlaminar delamination of the composite also include fiber–metal interfacial delamination, metal plastic deformation, and petal-shaped fracture.
4. Considering the flexibility in laminate structural design and complex practical engineering environments, this study has limitations: the high-velocity impact speed range is confined to 200–244.4 m/s. Furthermore, laminates in practical applications are subjected to impacts from projectiles of varying geometries at diverse incident angles. Comparative investigations on the impact resistance of laminates under multiple impact scenarios warrant further exploration. Future work could also integrate optimization design methods to conduct multi-objective optimization for laminates with additional stacking sequences, enabling comprehensive analysis of impact resistance across different layup configurations.

## Figures and Tables

**Table 1:** Performance parameters of T700 carbon fiber-reinforced PEEK prepreg.

Physical Property	Unit	Test Criteria	Test Result
Melting point of resin	°C	ISO 11357 [21]	343
Glass transition temperature of resin	°C	ISO 11357 [22]	143
Density	g/cm^3^	ASTM D792 [23]	1.62
Fiber surface density	g/cm^2^	ISO 10352 [24]	220
Fiber mass fraction	%	ASTM D3171 [25]	60
0° tensile strength	MPa	GB/T 1447-2005 [26]	2000
0° tensile modulus	GPa	GB/T 1447-2005	130
0° bending strength	MPa	GB/T 1449-2005 [27]	1600
0° bending modulus	GPa	GB/T 1449-2005	120
Interlaminar shear strength	MPa	JC/T 773-2010 [28]	120

**Table 2:** Test piece type.

Specimen Types	Thickness (mm)
[0/90/90/0]_5_	2.6
[0/90/90/0]_6_	3.12
[0/90/90/0]_5_/Al	3.6
[0/90/90/0]_6_/Al	4.12

**Table 3:** High-speed impact test results.

Specimen Types	Impact Speed (m/s)	Impact Energy (J)	Residual Velocity (m/s)	Residual Energy (J)	Energy Absorption (J)	Specific Energy Absorption (J·kg^−1^)	Energy Absorption per Unit Thickness (J·m^−1^)
[0/90/90/0]_5_	200.8	70.6	138.9	33.8	36.8	1553.1	14,153.1
[0/90/90/0]_6_	183.0	58.6	97.0	16.5	42.1	1482.2	13,506.4
[0/90/90/0]_6_	202.4	71.7	125.2	27.4	44.3	1556.7	14,185.5
[0/90/90/0]_5_/Al	202.4	71.7	69.0	7.1	64.5	1610.9	17,599.5
[0/90/90/0]_5_/Al	242.4	102.8	132.1	30.5	72.3	1838.0	20,080.0
[0/90/90/0]_6_/Al	200.4	70.3	-	-	-	-	-
[0/90/90/0]_6_/Al	244.4	104.5	106.0	19.7	84.9	1925.8	20,598.8

**Table 4:** Johnson–Cook model parameters for 2024-T3 [33,34,35].

Material Parameter	Quantitative Value	Model Parameter	Quantitative Value
ρ (g/cm^3^)	2.78	*n*	0.301
E (GPa)	73	*m*	1
ν	0.31	$D_{1}$	0.034
$T_{r}$ (K)	293	$D_{2}$	0.664
$T_{m}$ (K)	775	$D_{3}$	−1.5
A (MPa)	310	$D_{4}$	0.011
B (MPa)	456.4	$D_{5}$	0

**Table 5:** Parameters of finite element model for T700 carbon fiber-reinforced composite materials [37,38,39].

Material Parameter	Quantitative Value	Material Parameter	Quantitative Value
ρ (g/cm^3^)	1.62	$G_{23}$ (GPa)	3.6
$E_{1}$ (GPa)	130	$X_{t}$ (MPa)	2000
$E_{2}=E_{3}$ (GPa)	9.6	$X_{c}$ (MPa)	1360
$\nu_{12}=\nu_{13}$	0.37	$Y_{t}$ (MPa)	79
$\nu_{23}$	0.33	$Y_{c}$ (MPa)	176
$G_{12}=G_{13}$ (GPa)	5.7	$S_{c}$ (MPa)	130

**Table 6:** The error in the simulation results of residual velocity for projectiles.

Specimen Type	Impact Speed (m/s)	Simulation Result (m/s)	Simulated Energy Absorption (J/kg)	Error (%)	RMSE
[0/90/90/0]_5_	200.8	143.7	34.5	3.5	4.2
[0/90/90/0]_6_	183.0	104.8	39.4	8.0	8.2
[0/90/90/0]_6_	202.4	119.1	46.9	4.9	5.3
[0/90/90/0]_5_/Al	202.4	75.2	61.8	9.0	7.0
[0/90/90/0]_5_/Al	242.4	142.3	67.4	7.7	8.1
[0/90/90/0]_6_/Al	244.4	115.8	81	9.2	7.6

## Data Availability

The original contributions presented in this study are included in the article. Further inquiries can be directed to the corresponding author.

## References

[1] Asundi A., Choi A.Y. (1997). Fiber metal laminates: An advanced material for future aircraft. J. Mater. Process. Technol..

[2] Vogelesang L.B., Vlot A. (2000). Development of fibre metal laminates for advanced aerospace structures. J. Mater. Res. Technol..

[3] Sun J., Xu S., Lu G., Wang Q., Gong A. (2022). Ballistic impact experiments of titanium-based carbon-fibre/epoxy laminates. Thin-Walled Struct..

[4] Corderley G., Mostert F., Krüger J.J. (2019). Failure modes in a carbon/titanium fibre metal laminate under hyper-velocity impact. Int. J. Impact Eng..

[5] Zhu Z., Li X., Yang R., Xie W., Zhang D. (2023). The energy dissipation mechanism of bi-metal Kevlar\titanium fiber metal laminate under high-velocity impact. Eur. J. Mech.—A/Solids.

[6] Ali A., Pan L., Duan L., Zheng Z., Sapkota B. (2016). Characterization of seawater hygrothermal conditioning effects on the properties of titanium-based fiber-metal laminates for marine applications. Compos. Struct..

[7] Song Z., Ming S., Du K., Zhou C., Wang Y., Xu S., Wang B. (2022). Energy absorption of metal-composite hybrid tubes with a diamond origami pattern. Thin-Walled Struct..

[8] Wu G.Q., Pan Y.C., Zhang Z.K. (2016). Research progress on ultra-light fiber metal laminates. Aeronaut. Manuf. Technol..

[9] Rekatsinas C.S., Siorikis D.K., Nastos C.V., Chrysochoidis N.A., Theodosiou T.C., Yigit A.S., Christoforou A.P., Saravanos D.A. (2023). An efficient computational framework for hailstone impacts on composite plates utilizing a semi-empirical viscoplastic contact law. Int. J. Impact Eng..

[10] Zhang F.Q., Luo G., Zhang H.Y., Cong P., Liu L., Chen W. (2024). Experimental and numerical analysis study on the low and medium speed bird strike. Eng. Fail. Anal..

[11] Abdullah M.R., Cantwell W.J. (2012). The high-velocity impact response of thermoplastic-matrix fibre-metal laminates. J. Strain Anal. Eng. Des..

[12] Ferrante L., Sarasini F., Tirillò J., Lampani L., Valente T., Gaudenzi P. (2016). Low velocity impact response of basalt-aluminium fibre metal laminates. Mater. Des..

[13] Fatt S.M.H., Lin C., Revilock M.D., Hopkins D.A. (2003). Ballistic impact of GLARE™ fiber-metal laminates. Compos. Struct..

[14] Li X., Zhang X., Guo Y., Shim V., Yang J., Chai G.B. (2018). Influence of fiber type on the impact response of titanium-based fiber-metal laminates. Int. J. Impact Eng..

[15] Gao Y., Shi L., Lu T., Xie W., Cai X. (2024). Ballistic and delamination mechanism of CFRP/aluminum laminates subjected to high velocity impact. Eng. Fract. Mech..

[16] Li H., Li Z., Xiao Z., Wang X., Xiong J., Zhou J., Guan Z. (2021). Development of an integrated model for prediction of impact and vibration response of hybrid fiber metal laminates with a viscoelastic layer. Int. J. Mech. Sci..

[17] Yang Z.X., Wang D.K., Zhang C. (2024). Prediction and failure analysis of high-speed bird impact damage in fiber metal laminates. J. Mater. Sci. Eng..

[18] Yaghoubi A.S., Liaw B. (2012). Thickness influence on ballistic impact behaviors of GLARE 5 fiber-metal laminated beams: Experimental and numerical studies. Compos. Struct..

[19] Zhang L., Xie M.X., Zhang L.J., Zhang J.X. (2024). Research progress on interface regulation of fiber reinforced thermoplastic composites/metal connections. Welded Pipe Tube.

[20] Sheng L.Y., Lai C., Xu Z.F., Jiao J.K. (2019). Effect of the surface texture on laser joining of a carbon fiber-reinforced thermosetting plastic and stainless steel. Strength Mater..

[21] (2018). Plastics-Differential Scanning Calorimetry (DSC)-Part 6: Determination of Specific Heat Capacity.

[22] (2021). Plastics-Differential Scanning Calorimetry (DSC)-Part 2: Determination of Glass Transition Temperature.

[23] (2023). Standard Test Methods for Density and Specific Gravity (Relative Density) of Plastics by Displacement.

[24] (2017). Fibre-Reinforced Plastics—Moulding Compounds and Prepregs—Part 6: Determination of the Fibre Mass Content by Ignition Method.

[25] (2022). Standard Test Methods for Constituent Content of Composite Materials.

[26] (2005). Fiber-Reinforced Plastics Composites—Determination of Tensile Properties.

[27] (2005). Fibre-Reinforced Plastic Composites—Determination of Flexural Properties.

[28] (2010). Fibre-Reinforced Plastics Composites. Determination of Apparent Interlaminar Shear Strength by Short-Beam Method.

[29] Dong H.M., Yi X.S., An X.F., Zhang C.Q., Yan L., Deng H. (2014). Research progress on interlayer toughening of fiber reinforced thermosetting polymer matrix composites. J. Compos. Mater..

[30] Yun N.G., Won Y.G., Kim S.C. (2004). Toughening of carbon fiber/epoxy composite by inserting polysulfone film to form morphology spectrum. Polymer.

[31] Livingston R., Koohbor B. (2022). Characterizing fiber-matrix debond and fiber interaction mechanisms by full-field measurements. Compos. Part C Open Access.

[32] Johnson G.R., Cook W.H. A constitutive model and data for metals subjected to large strains, high strain rates and high temperatures. Proceedings of the 7th International Symposium on Ballistics.

[33] Zakeri M., Mansoori H., Sadeghian M., Guagliano M. (2022). Impact response of fiber metal laminates based on aluminum and UHMWPE composite: Numerical simulation. Thin-Walled Struct..

[34] Han J., Shi Y., Ma Q., Vershinin V.V., Chen X., Xiao X., Jia B. (2022). Experimental and numerical investigation on the ballistic resistance of 2024-T351 aluminum alloy plates with various thicknesses struck by blunt projectiles. Int. J. Impact Eng..

[35] Cheng J.C., Zhang S., Liu Q., Ye S., Luo S., Cai Y., Huang J. (2022). Ballistic impact experiments and modeling on impact cratering, deformation and damage of 2024-T4 aluminum alloy. Int. J. Mech. Sci..

[36] Kollár L.P., Springer G.S. (2003). Mechanics of Composite Structure.

[37] Hastie J.C., Guz I.A., Kashtalyan M. (2019). Effects of thermal gradient on failure of a thermoplastic composite pipe (TCP) riser leg. Int. J. Press. Vessel. Pip..

[38] Cherniaev A., Telichev I. (2015). Meso-scale modeling of hypervelocity impact damage in composite laminates. Compos. Part B Eng..

[39] Liu H.B., Liu J., Kaboglu C., Zhou J., Kong X., Blackman B.R., Kinloch A.J., Dear J.P. (2020). The behaviour of fibre-reinforced composites subjected to a soft impact-loading: An experimental and numerical study. Eng. Fail. Anal..

[40] Santiago R., Cantwell W., Jones N., Alves M. (2018). The modelling of impact loading on thermoplastic fibre-metal laminates. Compos. Struct..

[41] Li Z.-Y., Xue Y.-S., Sun B.-Z., Gu B.-H. (2023). Ballistic penetration damages of hybrid plain-woven laminates with carbon, Kevlar and UHMWPE fibers in different stacking sequences. Def. Technol..

[42] Shen Z., Hu D., Zhang Y., Cai Q., Han X. (2017). Continuous twice-impacts analysis of UHMWPE laminate fixed with bolted joints. Int. J. Impact Eng..

